# A microfluidic photobioreactor for simultaneous observation and cultivation of single microalgal cells or cell aggregates

**DOI:** 10.1371/journal.pone.0216093

**Published:** 2019-04-29

**Authors:** Christoph Westerwalbesloh, Carl Brehl, Sophie Weber, Christopher Probst, Janka Widzgowski, Alexander Grünberger, Christian Pfaff, Ladislav Nedbal, Dietrich Kohlheyer

**Affiliations:** 1 Institute of Bio- and Geosciences, IBG-1: Biotechnology, Forschungszentrum Jülich GmbH, Jülich, Germany; 2 Institute of Bio- and Geosciences, IBG-2: Plant Sciences, Forschungszentrum Jülich GmbH, Jülich, Germany; 3 Multiscale Bioengineering, Bielefeld University, Bielefeld, Germany; 4 RWTH Aachen University, Aachener Verfahrenstechnik (AVT.MSB), Aachen, Germany; Texas A&M University College Station, UNITED STATES

## Abstract

Microalgae are an ubiquitous and powerful driver of geochemical cycles which have formed Earth’s biosphere since early in the evolution. Lately, microalgal research has been strongly stimulated by economic potential expected in biofuels, wastewater treatment, and high-value products. Similar to bacteria and other microorganisms, most work so far has been performed on the level of suspensions which typically contain millions of algal cells per millilitre. The thus obtained macroscopic parameters average cells, which may be in various phases of their cell cycle or even, in the case of microbial consortia, cells of different species. This averaging may obscure essential features which may be needed for the correct understanding and interpretation of investigated processes. In contrast to these conventional macroscopic cultivation and measuring tools, microfluidic single-cell cultivation systems represent an excellent alternative to study individual cells or a small number of mutually interacting cells in a well-defined environment. A novel microfluidic photobioreactor was developed and successfully tested by the photoautotrophic cultivation of *Chlorella sorokiniana*. The reported microbioreactor facilitates automated long-term cultivation of algae with controlled temperature and with an illumination adjustable over a wide range of photon flux densities. Chemical composition of the medium in the microbioreactor can be stabilised or modulated rapidly to study the response of individual cells. Furthermore, the algae are cultivated in one focal plane and separate chambers, enabling single-cell level investigation of over 100 microcolonies in parallel. The developed platform can be used for systematic growth studies, medium screening, species interaction studies, and the thorough investigation of light-dependent growth kinetics.

## Introduction

Microalgae are a diverse group of photosynthetic organisms, including eukaryotic algae and prokaryotic cyanobacteria, which has formed our oxygen-containing atmosphere and remains responsible for a major fraction of carbon cycling which stabilises the Earth’s climate [1]. In addition to this immense ecological relevance, microalgae are also gaining importance as potential source of biofuels [2–4] and high value products, [5–7] or as tool to clean wastewater [8].

Microalgae are typically grown for biotechnological use in large-scale open cultivation systems [9] or in closed photobioreactors, [10] which often integrate monitoring of incident irradiance and real-time measurements of suspension parameters, e.g., optical density, pH, and temperature [11]. More key data, such as chlorophyll fluorescence or dissolved O_2_/CO_2_, are measured in real time in laboratory photobioreactors [12]. The culture is often further characterised by taking suspension aliquots and measuring in these samples, for example, chemical composition, dry weight and pigment content. These are mean parameters which typically represent several millilitres of the suspension with millions of algal cells contributing to the average.

An insight into culture heterogeneity that goes beyond suspension averages may be obtained by microscopic examination or by single-cell analysis devices such as flow cytometers, which may reveal multiple algal species or even heterotrophic bacteria or other pathogens [13, 14]. A unique opportunity to study algae in further detail offer microfluidic approaches that allow cells to grow and mutually interact under well-defined conditions while being simultaneously observed and analysed by microscopy in situ and in real time [15]. In such a microfluidic photobioreactor, individual algal cells can be observed in isolation or in small aggregates that may include separately identifiable species. As in any other photobioreactor, algal cells require medium which contains nutrients and dissolved CO_2_ or bicarbonate to support photosynthesis. Depending on the design and material either diffusion through the chip material or transport by the continuously perfused medium serves to supply CO_2_ and remove the O_2_ produced by photosynthetic water splitting. For their photoautotrophic growth, the cells also require photosynthetically active radiation in the range from 400 nm to 700 nm and a controlled temperature with an optimum often between 20 and 40°C.

Several microfluidic devices for algae cultivation have been reported so far: Au et al. have developed a digital microfluidics (DMF) system to cultivate *Cyclotella cryptica* inside aqueous microdroplets of 70 μL [16]. DMF is a lab-on-a-chip platform technology which enables the electrical manipulation of microdroplets between insulated electrodes. Au et al. applied online absorption measurements to their droplets and determined a growth rate comparable to that when cultivating *C. cryptica* in mL scale systems [16]. Shih et al. have also applied a DMF system cultivating *C. cryptica* inside 12 μL sized droplets with an illumination of up to 250 μmol/(m^2^ s) and have been able to demonstrate that *C. cryptica* produced more energy-rich lipids when illuminated at 580 nm, while a combination with 450 nm was favourable for growth and lipid production [17]. Aqueous droplets suspended in an oil phase have been applied for the cultivation of *Chlorella vulgaris* by Dewan et al. studying growth kinetics. During cultivation the droplets have been stored in a static array of droplet traps. With this device long-term cultivations up to one month have been achieved keeping a constant droplet size of 10 nL [18]. Lim et al. have developed a 3-step system for the cultivation, the induction and the sub sequential purification of lipids produced by *Chlamydomonas reinhardtii*. The algae have been cultivated inside a single chamber with 15 μL culture volume and a light intensity of 50 μmol/(m^2^ s). The lipid production has been induced simply by a medium change and for the final lipid extraction aqueous alcohols have been injected [19]. Kim et al. have designed a microfluidic cultivation system, where they have captured colonies of *Botryococcus braunii* in 64 trapping sites. They have implemented dynamic light changes and different intensities in each cultivation chamber to identify the best light dark cycle for *B. braunii* oil production by incorporating two additional dye layers, one for the intensity (up to 132 μmol/(m^2^ s)) and the second for the light-dark cycle [20]. Luke et al. have been able to develop a microfluidic long term cultivation system for a photoautotrophic cultivation of multiple organisms such as *C. sorokiniana* using a light source emitting up to 100 μmol/(m^2^ s) [21]. For *C. sorokiniana* a growth rate of 0.073/h has been obtained in the chip, compared to 0.056/h in shaking flasks. Apart from the one by Luke et al., all systems have tolerated cell growth on more than one layer, which complicates subsequent image analysis. Furthermore, self-shading effects within the colonies have impeded defined and homogeneous irradiation. Often not much technical information about the illumination method has been revealed although homogeneous and reproducible irradiation is central in algal cultivation. Recently microfluidic devices for algae cultivation have been reviewed comprehensively by Kim et al. [22].

In this article we present a microfluidic single-cell cultivation and analysis approach for algae and other photoautotrophic organisms particularly addressing monolayer cell growth and optimised irradiation. Therefore, a polydimethylsiloxane (PDMS) microfluidic cultivation chip was designed enabling single-cell analysis with spatio-temporal resolution and accurate control over irradiation, growth medium and temperature. A single device incorporates several hundred monolayer growth chambers ideally suited for algae cultivation. To facilitate photoautotrophic cultivation, we developed an innovative LED illumination unit, the size of a typical objective, which can be simply mounted into the motorised objective revolver of an inverted microscope. This setting facilitates fast and automated changes between algal illumination and time-lapse microscopy using a standard live-cell imaging microscope configuration. We successfully validated the performance of our approach with the photoautotrophic growth of *C. sorokiniana* at different light intensities. Furthermore, cross species interactions were observed demonstrating the versatile applicability.

## Materials and methods

### Silicon-SU8 mould

A silicon wafer serving as mould for the PDMS chip casting was fabricated under cleanroom conditions at the Helmholtz Nano Facility (HNF) [23]. Prior to the following thin film processing procedure, chromium-glass dark-field lithography photomasks were fabricated by HNF staff. More fabrication details including videos can be found in the publication by Grünberger et al. [24].

A 100 mm silicon wafer was cleaned with a Piranha solution and subsequently rinsed with DI water. Afterwards it was bathed in 1% hydrofluoric acid for 1 min and rinsed again with DI water. The wafer was then heated for 20 min at 200°C for dehydration. The silicon-SU-8 mould contained two stacked layers of photoresist. The first layer of SU-8 2002 of 4.5 μm (micro resist technology GmbH, Germany) photoresist was spincoated onto the wafer. Therefore, 1.5 mL of resist was applied and spincoated at 3800 rpm for 35 s. Then the wafer was softbaked at 65°C for 3 min followed by 3 min at 95°C. The photomask containing the cultivation chamber geometries (cultivation chambers) was aligned with the wafer at vacuum contact mode using a mask aligner (MA/BA Gen4 Pro Serie mit MO-Exposure Optic, SUSS MicroTec Lithography GmbH, Germany) and exposed to 350 nm 400 nm light with 700 mJ/cm^2^. Afterwards post exposure baking was performed (3 min at 65°C and 3 min at 95°C). The wafer was placed in SU-8 developer for 1 min and transferred into a second container with fresh SU-8 developer for a few seconds. Then the wafer was rinsed in isopropanol to remove any SU-8 developer residue and dried using nitrogen gas flow. Afterwards a hard bake was performed for 10 min at 150°C. The second SU-8 layer of 10 μm SU-8 was spincoated onto the wafer at 2600 rpm for 35 s. The coated wafer was softbaked for 3.5 min at 65°C and 3 min at 95°C. The second layer photomask incorporating the geometries for the medium supply channels was aligned in the mask aligner with the first layer mask and exposed to 350 nm 400 nm light with 800 mJ/cm^2^ in vacuum contact mode. Afterwards, a SU-8 post exposure bake was performed on a hotplate for 3 min at 65°C and 3 min at 95°C. The wafer was again placed in SU-8 developer for 45 s and transferred into a second container with fresh SU-8 developer for 60 s. To remove any SU-8 developer residue the wafer was rinsed 20 s with isopropanol and dried with nitrogen gas. Finally, a hard bake was performed for 6 h at 150°C. The finished wafer was used as master mould for PDMS moulding. To validate the structure heights profilometer measurements (Dektak 150, Veeco, USA) were made. Finally, the accuracy of the structures was controlled optically using a microscope.

### Microfluidic chip moulding and assembly

PDMS chips were fabricated by preparing 60 g of a 10:1 mixture of the polymer base and cross linker (Silicone Elastomer Kit #184, Dow Corning, Midland), which was carefully mixed and degassed in a desiccator (Duran, Germany) at 200 mbar for 30 min. When the mixture was transparent and without any air bubbles it was poured onto the SU-8 mould in a petri dish and crosslinked at 80°C for 1 h on a levelled plate inside a laboratory oven (UN 200, Memmert, Germany). The height of the hardened PDMS layer was typically between 3 mm 4 mm. After baking, the PDMS was removed from the mould, cut into single chips and inlets and outlets were punched using a punching tool (Uni-Core, D = 1 mm, Fisher Scientific, Germany). To remove particles the chips were rinsed with isopropanol and dried under nitrogen gas flow. Remaining particles were removed by reversibly applying duct tape to the PDMS surface. All working steps were performed under a crossflow clean bench to reduce contamination with dust particles. The chip was subsequently placed in a plasma generator together a glass slide of 170 μm thickness serving as the glass bottom, and both parts were oxygen plasma treated for 25 s (Femto Plasma Cleaner, Diener Electronics, Germany) at 0.8 mbar with a gas flow of 15 sccm. Immediately afterwards the PDMS chip was placed carefully on the glass slide to initiate bonding. At last, the PDMS-glass chip was baked at 80°C for 2 min to strengthen the bonding.

### Microfluidic cultivation

Algae cells were randomly inoculated into the cultivation chambers by infusing the cell suspension so that cells got slightly stuck inside the shallow cultivation chambers whose height matches the diameter of an average cell. Under cultivation conditions a homogeneous medium flow with equal flow rates leads to cultivation chambers with predominantly diffusive mass transport. However, the primary inoculation procedure requires unequal volume flows in the medium supply channels adjacent to the chambers resulting in convection through the cultivation chambers. Therefore, flow conditions can be temporarily distorted by an air bubble entrapped inside the microfluidic channels as has been described in detail by Probst et al. [25]. 1 mL of the preculture was filled in a syringe to load the cells into the cultivation chambers. After the chip was loaded with cells the tubing of the medium containing syringes was connected to the inlets of the chip and the outlets were connected to waste containers. Cultivation medium was pumped with a flow rate of 200 nL/min per syringe through the supply channels by a syringe pump (neMESYS, Cetoni, Korbussen, Germany).

Experiments were conducted with an inverted time-lapse microscope (Eclipse Ti-E, Nikon, Japan). For all cultivations, a 40x air objective was used (Plan Apo 40X/0.95 DIC, Nikon, Japan). The microscope was heated using an incubation chamber at 30°C (Temp-Controller 2000-2, Pecon, Germany), cultivation took place under ambient CO_2_ concentration. Images were taken with a CCD colour camera (DS-Fi, Nikon, Japan) using the Nikon software NIS Elements AR 4.40.02. Cell growth was recorded by sequential time-lapse imaging of up to 80 chambers every 30 min. For growth studies only the chambers containing a single starting cell were chosen for further analysis.

### Illumination unit

**Fig 1:**
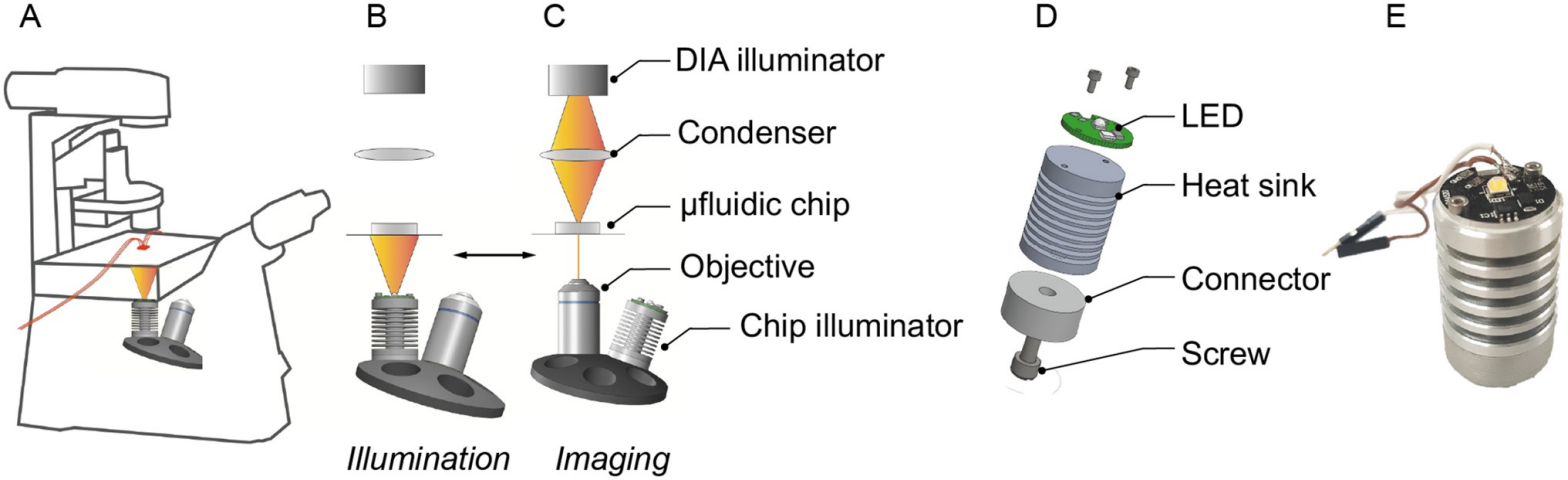
Microscopy and illumination setup. (A) Inverted microscope with the inserted chip and connected tubing (red). (B) Objective revolver with inserted LED illumination device in illumination mode and (C) in imaging mode. (C) LED below the chip for illumination. (D) Exploded assembly drawing of the LED with the heat sink and connector. (E) Photograph of the assembled lighting unit.

The illumination unit (Fig 1D) incorporates a warm white LED with 125° emission (MWWHD3—3000 K, 2000 mW LED on a metal-core PCB, 700mA, Thor Labs, USA). The emission spectrum can be found in S1 Fig. The PCB is mounted onto an in-house fabricated aluminium cooling unit fixed to an acrylic glass thread matching the specifications of conventional Nikon objectives. The illumination unit was installed inside the objective revolver replacing a conventional objective. The electrical connection to the LED has to be positioned with care and 360° rotations of the revolver must be avoided to minimise the risk of a detached wiring. (see Fig 1D). The LED irradiance intensity was controlled by an LEDD1B T-Cube LED Driver (Thor Labs, USA), which was governed by an USB-6008 DAQ device (National Instruments, USA). The illumination was controlled via macros (see S2 and S3 Files) implemented into the Nikon NIS Elements software in such a way, that the LED was automatically switched off when the objective revolver changed to the desired objective and time-lapse imaging was initiated.

To determine the irradiation intensity a LI-190R Quantum Sensor connected to an LI-250A measurement device (LI-COR, USA) was applied. This device measures the power within the PAR wavelength band between 400 nm and 700 nm, thereby the irradiation provided for photoautotrophic organism can be determined independently of the light source and is typically given in μmol/(m^2^ s) [26, 27]. The sensor was placed in a standard specimen tray holder and a 170 μm glass substrate was placed in the sensor spot.

Temperature measurements were performed using an MTC-20/2SD temperature controller equipped with a TS-200 miniature sensor (npi electronic GmbH, Tamm, Germany). Therefore, the TS-200 miniature sensor was incorporated into a PDMS chip in close proximity to the cultivation chambers.

### Strain, media and precultivation

*Chlorella sorokiniana* 211-8k, was obtained from the culture collection in Göttingen, Germany [28]. The precultures were inoculated from a Tris-Acetate-Phosphate nutrient medium (TAP) agarose plate and cultivated in TAP medium for 20 h at 30°C in Erlenmeyer flasks [29, 30]. For the microfluidic experiments TP medium was used, which is TAP without acetate. The pH was adjusted to pH 7 using HCl and the medium was autoclaved and stored at 4°C. The TAP medium was mixed for all experiments as follows in water: 20 mmol/L Tris(hydroxymethyl)-aminomethan (H_2_NC(CH_2_OH)_3_), 7 mmol/L NH_4_Cl, 0.83 mmol/L MgSO_4_ ⋅ 7H_2_O, 0.45 mmol/L CaCl_2_ ⋅ 2H_2_O, 1.65 mmol/L K_2_HPO_4_, 1.05 mmol/L KH_2_PO_4_, 0.134 mmol/L Na_2_EDTA ⋅ 2H_2_O, 0.184 mmol/L ZnSO_4_ ⋅ 7H_2_O, 40 μmol/L H_3_BO_3_, 32.9 μmol/L MnCl_2_ ⋅ 4H_2_O, 12.3 μmol/L FeSO_4_ ⋅ 7H_2_O, 10 μmol/L CoCl_2_ ⋅ 6H_2_O, 10 μmol/L CuSo_4_ ⋅ 5H_2_O, 4.44 μmol/L (NH_4_)_6_MoO_3_ and 1 mL Ch_3_COOH. If not otherwise stated, all chemicals were purchased from Carl Roth, Germany.

### Growth rate determination

Computer aided image analysis was performed to quantify cell growth. Therefore, the projected surface area occupied by the algae colonies was automatically determined with the “Auto Threshold” plugin in ImageJ (Version 2.0.0) by first thresholding an 8-bit image and then filling the areas between the cells with the “Binary plugin”. The process was written as jython-script for ImageJ (see S1 File). Afterwards, the detected area was optically inspected and mismatches were manually corrected. The area over time was used from the start of the experiment until the chamber was filled with cells and excess cells were lost due to convective flow in the supply channel. This covered time spans depending on the light intensity. The corresponding growth rate was then calculated by applying an exponential fit to the determined cell area plotted over time using Origin (ORIGIN 9.1, OriginLab Corporation, USA).

## Results and discussion

### Cultivation concept

The present system was designed to allow the long term cultivation of single microalgae and clusters under well controlled environmental conditions, considering medium, irradiation, and temperature, typically starting from the inoculum of a single cell.

**Fig 2:**
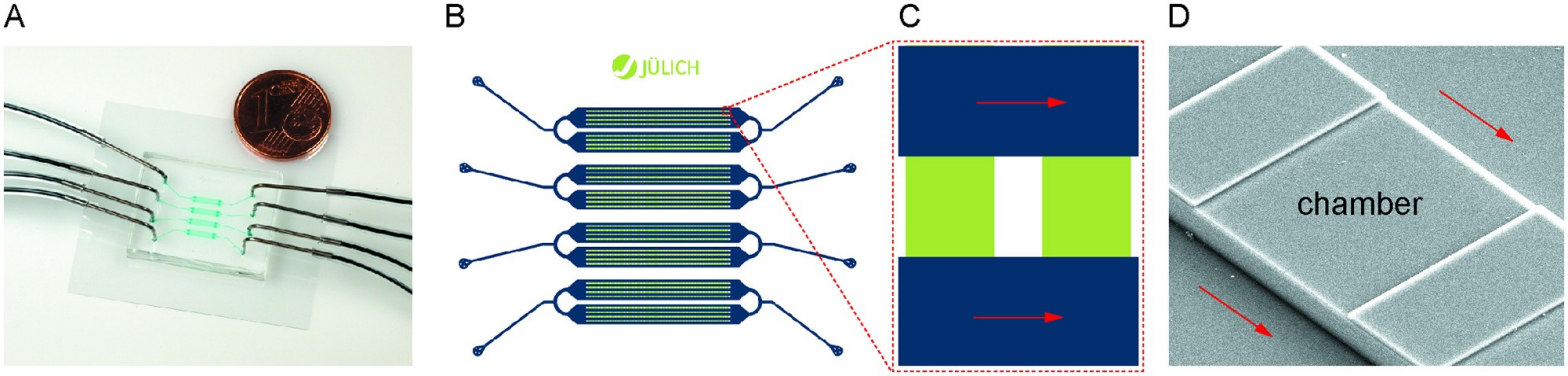
Microfluidic single-cell cultivation platform used in this study. (A) Photograph of the assembled microfluidic chip. (B) Overview over the four channels on each chip. (C) Detail of the cultivation chambers (green, dimensions 80 μm × 90 μm, height 4.5 μm) between two supply channels (blue, width 100 μm, height 15 μm), red arrows show cultivation medium flow. (D) SEM image of the PDMS chip showing one chamber.

To meet the requirements for unbiased algae growth, the dimensions of the cultivations chambers by Grünberger et al., which have originally been developed to accommodate smaller organisms, for example *Escherichia coli* or *Corynebacterium glutamicum*, were modified [31]. The limited chamber height of now 4.5 μm confines all microalgae to grow within a single-cell monolayer. This configuration is ideal for microscopy and subsequent image analysis to derive data with spatial and temporal resolution, and furthermore eliminates cellular self-shading. The chip (2.2 cm × 1.7 cm) contains 4 × 8 × 50 cultivation chambers, each of them 4.5 μm high and 80 μm × 90 μm big, between supply channels of 15 μm height and of 100 μm width (see Fig 2). Each of the four main channels has an independent media inlet and supply outlet to permit parallel experiments with regard to media composition. With the chosen flow rate of 200 nL/min the average flow velocity within the supply channels is 0.44 mm/s. This means the volume flowing by the chamber within a second is approximately 100 times the chamber volume, therefore granting excellent control of medium conditions within the chambers since new nutrients are supplied and any metabolic products are carried away. The flow also replaces the medium in the chip completely within less than 1 min, enabling fast changes in medium conditions if required. We were able to inoculate a portion of the chambers with a single cell each and the resulting colonies remained spatially separated over the whole course of the experiment. Observation of over five generations of *C. sorokiniana* (over 600 cells) was possible before cell loss due to wash out occurred.

The illumination system is crucial for the success and reproducibility of photoautotrophic cultivations. Earlier experiments using our microfluidic cultivation system, applying either the integrated bright field microscope lamp or an external LED placed above the PDMS-chip, could not meet required standards. The microscope illumination is typically not well suited for simultaneous high contrast microscopy imaging and algae irradiation. When the external LED was used, it was placed between the PDMS chip and the condenser lens and interfered with microscopic illumination. Furthermore, variations in the PDMS thickness differences led to light intensity variations. Therefore, we developed a innovative LED illumination unit, the size of a typical objective, which can be simply mounted into the motorised objective revolver of an inverted microscope. This setting facilitates fast and automated changes between independent algal illumination and time-lapse microscopy using a standard inverted live-cell imaging microscope illumination as shown in Fig 1 (see S5 Video).

The LED performance was validated by measuring the illumination levels at the glass bottom below the chip. The minimum level was 19 μmol/(s m^2^) and the maximum level was 1941 μmol/(s m^2^). While the current controlling the light intensity emitted by the LED can be modulated freely, we found in our experiments a minimum step width of approximately 20 μmol/(s m^2^) easily attainable for a precise control of the illumination level. The undesired heat emitted by the LED was sufficiently dispersed by the aluminium cooling unit, so that the temperature in close proximity to the cultivation chambers did not exceed (31.8 ± 0.5)°C for the highest intensity, and for 19 μmol/(s m^2^) and 110 μmol/(s m^2^) (30.1 ± 0.1)°C and (30.3 ± 0.2)°C respectively (5 measurements each). At the same time the acrylic connector minimised heat conduction towards the microscope as it has a poor thermal conductivity. Furthermore, the LED position below the cultivation chip enabled reproducible irradiation within technical replicates since the glass cover of the cultivation chip did not vary significantly. We measured the intensity in the centre and at the four corners of the cultivation area of approximately 4.3 mm × 6.9 mm and found lower intensities at the corners than in the centre, but not lower than 93% of the centre value. This shows the good spacial homogeneity of the implemented illumination solution. The thread of the LED-unit can be modified easily to be compatible with different microscopy standards, therefore supporting most use cases.

### Biological validation

*C. sorokiniana* was chosen as a model organism to validate our cultivation and illumination system. The present strain was selected due to its good growth at high temperatures [32] and potential for industrial applications [33]. We tested the functionality of the system via the photoautotrophic cultivation at different irradiation levels. For this purpose, three different intensities were chosen (19, 110 and 1941 μmol/(s m^2^)). The three intensities are the lowest possible intensity the LED could emit, an intensity close to the magnitudes common in microfluidic algae cultivations, and the maximum intensity of the used LED. During our experiments we determined and analysed the growth rates, the splitting pattern and the interaction of the algae with a parasitic contamination. We have not investigated specifically the viability of cells trapped and cultivated in the device. However, most of the observed cells grew over the whole time of the experiment, so that we expect no major influence of the cultivation device on viability.

**Fig 3:**
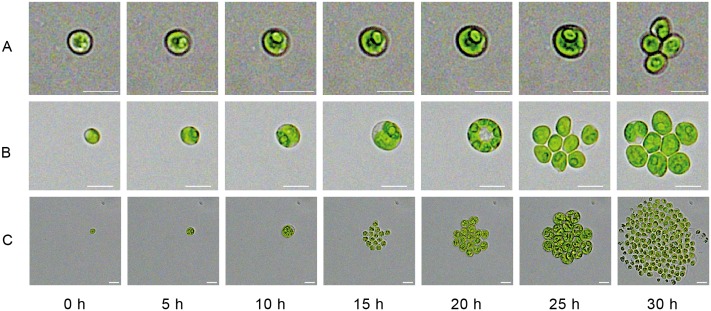
Examples of growth for different light intensities. Scale bar is always 5 μm. (A) For a light intensity of 19 μmol/(s m^2^) the average observed growth rate was 0.041/h, (B) at an intensity of 110 μmol/(s m^2^) it was 0.084/h, and (C) for light intensity of 1941 μmol/(s m^2^) it was 0.109/h. For the purpose of illustration the figure contrast was enhanced.

The growth of three exemplary colonies at the chosen intensities can be seen in Fig 3 (see also S1–S3 Videos). At the lowest intensity we determined a growth rate of (0.041 ± 0.003)/h over 140 h of cultivation time for 3 microcolonies, for 110 μmol/(s m^2^) a growth rate of (0.084 ± 0.006)/h over 50 h of cultivation time for 4 microcolonies and at high light intensities (1941 μmol/(s m^2^)) a growth rate of (0.109 ± 0.031)/h over 30 h of cultivation time for 7 microcolonies. The cultivation time differs because the chambers filled faster with cells for the high light intensities, and as soon as cells started to leave the chambers the growth rate determination had to be stopped. Sorokin has applied a lab-scale cultivation system and has reported a growth rate of 0.17/h for the cultivation of *C. sorokiniana* at 30°C [32]. The higher growth rates could be explained by a more efficient gas exchange within the large-scale cultivation system since active gassing enhances CO_2_ availability and has not been used for the microfluidic cultivation. It should also be noted that we determine the growth rate from colony areas and not from cell dry weight or optical density. The method based on area works best for big cultures with homogeneous cell size distributions and less well for single cells. Our results show that the presented microfluidic device is very suitable for algae cultivation with comparable growth rates as they have been reported for conventional scales of cultivation in free suspension, although there is no height restriction to growth like in the microfluidic chamber. Using a microfluidic cultivation approach, only Luke et al. have cultivated cells of *C. sorokiniana* in a cellular monolayer [21]. In agreement with our findings, they have reported a similar growth rate of 0.073/h for cultivation in BG11 medium.

Monolayer cultivations in isolated chambers support accurate identification and tracking of single cells and clusters with spatio-temporal resolution, enabling systematic analyses of the cellular division process, morphology etc. At low light intensities the mother cells typically divided into four new cells, at medium intensity into eight cells and the highest possible illumination the algae mainly formed 16 new daughter cells (see Fig 3). This division pattern is similar to the closely related algae *C. vulgaris*, which has been reported to split into 2 to 32 daughter cells [34, 35]. The amount of newly formed daughter cells of *C. sorokiniana* further depends strongly on other conditions affecting cells growth, for example the availability of nutrients [34]. Microfluidic experiments with single-cell resolution can help to understand the interaction of the different factors and how their combination results in specific growth behaviour.

**Fig 4:**
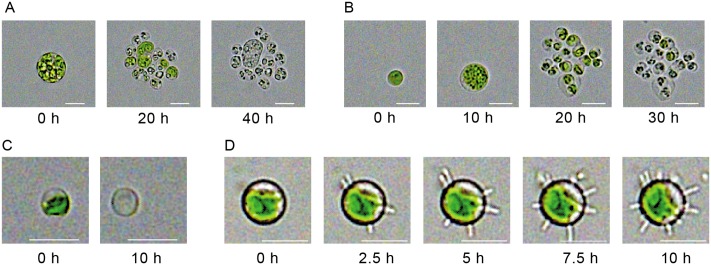
(A, B, C) Examples of cell death at high light intensity (1941 μmol/(s m^2^)). (D) Infection of algae with a parasite. Scale bar is always 5 μm. For the purpose of illustration the figure contrast was enhanced.

During experiments with the maximum irradiation (1941 μmol/(s m^2^)) a severe impact of light could be observed for some cells. Instead of faster growth, some cells did not grow any further and typically lysed (see Fig 4A, 4B and 4C). This phenomenon may have been caused by photoinhibition of Photosystem II reaction centres which occurs under high light conditions when the D1-protein damage exceeds repair capacity [36]. For several cells we observed cell death soon after inoculation, when they were exposed to the high light intensity for the first time (Fig 4C). Other cells did grow and divide, before the next generation of cells stopped growing and the chloroplasts started to bleach out as clearly indicated by the green colour fainting away (Fig 4A and 4B). Nevertheless, chambers containing cell debris from lysed cells were not used for the automated evaluation, as cell fragments got stuck in the chamber and interfered with the detection algorithm.

The presented cultivation system also showed great scientific potential for the investigation of cell consortia, including for example bacteria and algae. During some of our cultivations an unexpected but interesting contamination occurred. We were able to observe the cellular interaction between the micro algae *C. sorokiniana* and a vital parasitic bacterium over the course of several hours (see Fig 4D, see also S4 Video). The observed organism could be the *Vampirovibrio chlorellavorus*, a known parasite in algae cultures [37]. As the growth medium was sterile filtered before use, we expect the contamination to stem from a contaminated preculture and to have entered the chip during the seeding procedure. The contamination could be observed in all chambers of a contaminated lane on the chip. A possible countermeasure would be the application of pH-shocks [38], but this has not been tried within this study because the contamination was not observed in the relevant growth experiments.

## Conclusions

The presented microfluidic cultivation system for photoautotrophic cultivation of microalgae, with single-cell resolution under precisely controlled conditions regarding illumination, medium supply and temperature, was tested with the green algae *C. sorokiniana* under different light conditions. We were able to show how the system allows to investigate the influence of light on growth, division behaviour and cell death. The system facilitates future studies imitating light dark cycles as they can be found in nature or more complex dynamic light conditions found in macroscopic photo bio reactors, helping to verify computational models [39] and simplify the scale-up process [40]. As the system is coupled to an inverted microscope, other studies could make use of fluorescent bio markers to investigate population heterogeneity. The excellent medium control could help screen for optimal nutrient conditions. It also showed promise for the analysis of mixed cultures, in this case specifically parasite-algae interactions. The presented systems allows to gain a deeper understanding of algae bioprocesses at lab- and large-scale, via cultivation and observation with well-defined illumination and medium composition.

## Supporting information

S1 Fig Illumination spectrum. The spectrum from the LED as found in the manufacturers Data-sheet.
(TIF)

S1 File Image analysis script. The jython-script has been used within fiji to detect boundaries of the algae colonies.
(PY) Click here for additional data file.

S2 File Macro to switch LED off. There are two macros, one before imaging and one after, which were executed via Nikon NIS Elements, to control the illumination and positioning of the objective revolver (“Nosepiece”). The LED on/off switch was implemented as additional “Shutter”.
(TXT)

S3 File Macro to switch LED on. There are two macros, one before imaging and one after, which were executed via Nikon NIS Elements, to control the illumination and positioning of the objective revolver (“Nosepiece”). The LED on/off switch was implemented as additional “Shutter”.
(TXT)

S1 Video Growth at low intensity. (AVI)

S2 Video Growth at normal intensity. (AVI)

S3 Video Growth at high intensity. (AVI)

S4 Video Growth with contamination. (AVI)

S5 Video Light source switch. (AVI)
