# Supplementary figures and images for: A microfluidic photobioreactor for simultaneous observation and cultivation of single microalgal cells or cell aggregates
Source: PLoS One. 2019 Apr 29;14(4):e0216093. doi: 10.1371/journal.pone.0216093 (PMC6488086; doi:10.1371/journal.pone.0216093)

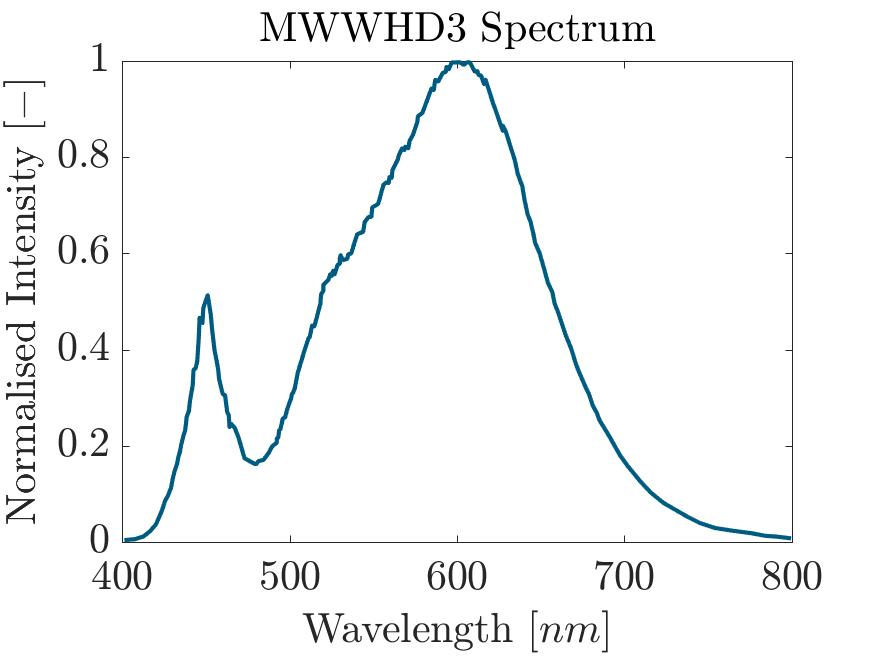

Supplement: S1 Fig — The spectrum from the LED as found in the manufacturers Data-sheet. (TIF) [file pone.0216093.s001.tif]
